# The Transgenerational Transmission of the Paternal Type 2 Diabetes-Induced Subfertility Phenotype

**DOI:** 10.3389/fendo.2021.763863

**Published:** 2021-11-05

**Authors:** Eva Zatecka, Romana Bohuslavova, Eliska Valaskova, Hasmik Margaryan, Fatima Elzeinova, Alena Kubatova, Simona Hylmarova, Jana Peknicova, Gabriela Pavlinkova

**Affiliations:** ^1^ Laboratory of Reproductive Biology, Institute of Biotechnology Czech Academy of Sciences (CAS), Biotechnology and Biomedicine Center of the Academy of Sciences and Charles University in Vestec (BIOCEV), Vestec, Czechia; ^2^ Laboratory of Molecular Pathogenetics, Institute of Biotechnology Czech Academy of Sciences (CAS), Biotechnology and Biomedicine Center of the Academy of Sciences and Charles University in Vestec (BIOCEV), Vestec, Czechia; ^3^ Department of Internal Medicine, Second Faculty of Medicine, Charles University in Prague and Motol University Hospital, Prague, Czechia

**Keywords:** sperm, diabetes, testes, fertility, offspring, molecular biomarkers, TERA, GAPDS

## Abstract

Diabetes is a chronic metabolic disorder characterized by hyperglycemia and associated with many health complications due to the long-term damage and dysfunction of various organs. A consequential complication of diabetes in men is reproductive dysfunction, reduced fertility, and poor reproductive outcomes. However, the molecular mechanisms responsible for diabetic environment-induced sperm damage and overall decreased reproductive outcomes are not fully established. We evaluated the effects of type 2 diabetes exposure on the reproductive system and the reproductive outcomes of males and their male offspring, using a mouse model. We demonstrate that paternal exposure to type 2 diabetes mediates intergenerational and transgenerational effects on the reproductive health of the offspring, especially on sperm quality, and on metabolic characteristics. Given the transgenerational impairment of reproductive and metabolic parameters through two generations, these changes likely take the form of inherited epigenetic marks through the germline. Our results emphasize the importance of improving metabolic health not only in women of reproductive age, but also in potential fathers, in order to reduce the negative impacts of diabetes on subsequent generations.

## Introduction

Type 2 diabetes mellitus (T2D) combines insulin resistance and insulin secretion deficiency with the main characteristics of carbohydrate, protein, and lipid metabolism disorders (1, 2). The progression of the disease is clinically manifested by increasing weight gain, impaired glucose tolerance, and high plasma insulin, triglycerides, and fasting glucose. The worsening glycemic control causes the development of long-term complications, including diabetic retinopathy, nephropathy, peripheral neuropathy, autonomic neuropathy, and increases risk of cardiovascular disease (2). T2D is also an established risk factor for sexual dysfunction in both men and women (3). In men, diabetes is associated with erectile dysfunction, androgen deficiency, and disruption of the hypothalamic-pituitary-gonadal axis (4–6). Additionally, clinical data from IVF clinics show that type 1 and type 2 diabetic male patients have lower *in vitro* fertilization success rates, suggesting that diabetes-exposed sperm are damaged, including increased sperm nuclear DNA fragmentation, altered sperm morphology, and reduced sperm motility (7–10). With the increasing prevalence and incidence of T2D, more men in their reproductive years will be affected, contributing to the increasing prevalence of subfertility. However, the molecular mechanisms responsible for diabetic environment-induced sperm damage and overall decreased reproductive outcomes are not fully established.

In addition, parental exposure to the diabetic environment represents a higher risk of adverse effects for the offspring, influencing effects on their cardiovascular and metabolic health, and increased susceptibility to metabolic and cardiovascular disorders later in life (11–18). The epidemiological evidence is supported by experimental animal studies that demonstrate the transmission of diabetic phenotypes to the offspring (19–24). The effects of maternal exposure to diabetes combine the direct effects of *in utero* exposure, genetic effects (nuclear and mitochondrial DNA), and epigenetic modifications of germ cells. Metabolic phenotypes can also be transmitted *via* the paternal lineage. Paternal effects that result from environmental exposures represent predominantly epigenetic modifications in sperm, although particular environmental factors can affect the composition of seminal fluid, which can produce placental and developmental effects (25). For example, high fat diet exposure of male rats reprograms ß cells in offspring (26), high-fat diet-induced paternal obesity modulates the sperm microRNA content and DNA methylation status (27), and paternal T2D alters DNA methylation patterns in sperm, involving changes in methylation of insulin signaling genes (28).

Although the molecular mechanisms associated with the transmission of the diabetic phenotype to the offspring have been investigated, the impact of paternal T2D exposure on the reproductive outcomes of subsequent generations remains unclear. Previously, we showed that paternal type I diabetes induced transgenerational changes in the testes, and increased sperm damage in the offspring across two generations (29). Here, we describe T2D exposure effects on the reproductive system and the reproductive outcome of fathers and their offspring, using a mouse model. This is the first complex analysis of the metabolic and reproductive system phenotypes of TD2 fathers and their descendants, demonstrating how paternal exposure to TD2 mediates intergenerational and transgenerational effects on the reproductive health of the offspring.

## Materials & Methods

### Experimental Animals

**Figure 1:**
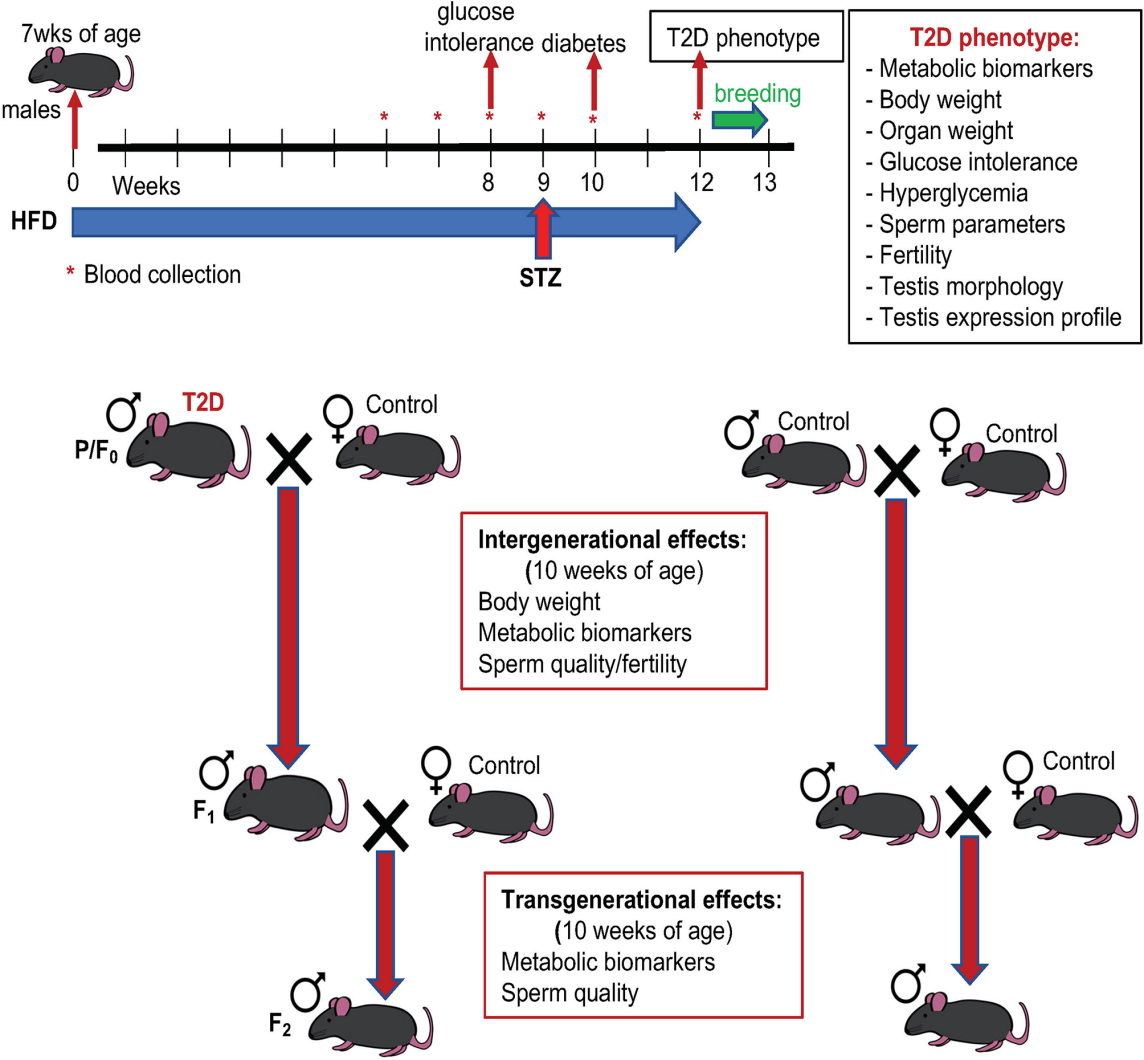
Experimental schematics. Type 2 diabetes (T2D) was induced by a combination of high fat diet (HFD) and a low dose of streptozotocin (STZ): sexually matured males (7 weeks old) were kept on HFD for 12 weeks before mating and injected with STZ at week 9 of the HFD experiment. T2D males and standard diet (control) fed males (parental generation, P/F_0_) were mated with standard diet fed females. After one week mating pairs were separated, males were killed, and tissues were collected for further analyses. The F_1_ male offspring were mated at 9 weeks of age, after one week mating pairs were separated. F_1_ males were killed, and analyses were performed at 10 weeks of age. Effects of diabetic paternal environment was also evaluated in the second (F_2_) male offspring generation at 10 weeks of age. Both F_1_ and F_2_ males were maintained on standard diet.

This study was conducted in accordance with the Guide for the Care and Use of Laboratory Animals (NIH Publication No. 85-23, revised 1996). The experimental protocol was approved by the Animal Care and Use Committee of the Institute of Molecular Genetics, CAS and carried out in accordance with the relevant guidelines and regulations. We used a T2D mouse model generated by a combination of high-fat diet and a low dose of streptozotocin (28, 30). Male inbred C57BL/6J mice (purchased from Charles River, Germany), aged 6 weeks, were divided into two groups and were fed either a high-fat diet (HFD: 60% of the metabolizable energy coming from fat, 20% from carbohydrates, and 20% from protein; ssniff^®^ EF acc. D12492 from Ssniff Spezialdiäten GmbH, Germany) or a control standard chow diet (11% of the metabolizable energy coming from fat, 65% from carbohydrates, and 20% from protein, #1320 diet, Altromin, Germany). After 9 weeks of HFD, intraperitoneal injection of 100 mg/kg body weight of streptozotocin (STZ; Sigma) was applied. Body weight, and blood samples were taken after 6-h fasting at indicated time points during the experiment (Experimental schematics in **Figure 1**). Blood glucose levels were measured in animals by a glucometer (COUNTOUR TS, Bayer, Switzerland); blood glucose levels maintained above 13.9 mmol/L are classified as diabetic. Mice were kept under standard experimental conditions with constant temperature (23–24°C). Glucose tolerance tests (GTT) were performed in 6h-fasted males. Mice were injected intraperitoneally with glucose (2 g/kg). Blood glucose was measured at 0, 15, 30, 60, and 120 min after glucose administration. After 12 weeks in the experiment, T2D males were glucose intolerant with blood glucose levels maintained above 13.9 mmol/L.

At 18 weeks of age, male founders (P generation) were mated with females fed with the standard chow diet. Individual males were placed in a cage with 1 healthy adult female. The animals were kept together for a week with minimalized disruptions and therefore, a vaginal plug was not checked in female mice. During this week all mice were fed with a standard chow diet. Immediately after separation, the males were killed, tissues and blood were collected for analyses. The females were housed individually during the gestation period and the litter size was recorded. Only male pups, the F_1_ offspring, were left in the litters to be used for following up transgenerational studies. At 9 weeks of age, randomly selected F_1_ males were mated with females to produce the F_2_ offspring. The offspring were fed only by the regular control diet. To minimize any additional environmental influence, molecular and cellular analyses were performed in both F_1_ and F_2_ male offspring at 10 weeks of age.

### Biochemical Parameters

Blood serum was collected from non-diabetic and T2D males following a 6-h fast and was analyzed using a fully automated chemistry analyzer, Beckman Coulter AU480 (Beckman) according to the manufacturer’s protocol in the Core Facility for Phenogenomics, Biocev. System Beckman Coulter AU480 reagents used for the quantification: glucose (OSR6621), AST (OSR6009), ALT (OSR6007), TG (OSR60118), insulin (33410), PAI-1 (plasminogen kit 20009000), and LDL (ODC0024).

### Sperm Parameters

Mouse sperm cells were obtained from the left and right cauda epididymis. Spermatozoa from both caudae were left to release into warm (37 °C) PBS in a CO_2_ incubator for 15 min. The resulting mixture was poured through a 30 μm filter (Partec) to obtain only the sperm fraction and PBS was added to 1 mL of final volume (31). Sperm were counted in a Bürker chamber under the light microscope, and sperm concentration, sperm morphology, and sperm head separation were determined as described previously (32). Sperm morphology was evaluated in 200 cells per each animal. Sperm viability was analyzed with SYBR14 from Live/Dead sperm viability kit (Molecular Probes, USA) and by flow cytometry (LSRII, blue laser 488 nm, Becton Dickinson, USA), minimum 15,000 events were evaluated. An annexin V–FITC apoptosis kit (Sigma) and chromomycin A_3_ staining (CMA3, Sigma) were used to assess the sperm damage (29). The molecular and functional quality of sperm was further evaluated by the expression of two molecular biomarkers, sperm acrosomal transitional endoplasmic reticulum ATPase (TER ATPase, TERA) and glyceraldehyde-3-phosphate dehydrogenase-S (GAPDS), using monoclonal antibodies Hs-14 (33) and Hs-8 (34), respectively (EXBIO Ltd., Czechia). These antibodies are used for pathological human sperm and reproductive diagnostics, and evaluation of dysfunctional spermatogenesis (33, 35–37). A minimum of 200 spermatozoa per each animal was examined with a Nikon Eclipse E400 fluorescent microscope equipped with a Nikon Plan Apo VC 60/1.40 oil objective (Nikon Corporation Instruments Company) and photographed with a ProgRes MF CCD camera (Jenoptik, Germany) with the aid of NIS-ELEMENTS imaging software (Laboratory Imaging, Ltd.). Protamine 1 and 2 ratio was obtained from 4 x 10⁶ sperm cells per each sample, as described previously (29).

### Morphological and Immunohistochemical Analyses of Testes

Dissected testes were fixed with 4% paraformaldehyde in PBS (pH 7.4) at 4°C overnight, and embedded in paraffin. Paraffin-embedded tissues were cut into 8 µm sections, de-paraffinized and rehydrated sections were stained with hematoxylin & eosin or used for immunohistochemistry. Citrate buffer (pH 6.0) was used as a heat-induced antigen retriever. Evaluation of meiotic cells and the 12 stages of production of spermatozoa in the mouse seminiferous epithelium was done using immunolabeling of the axial element of the synaptonemal complex (SYCP3, mouse anti-SCP3 #ab97672, 1:1000 dilution, Abcam). Rabbit anti-CX43 (#C6219, 1:2000 dilution, Sigma) was used to evaluate gap junctions. The secondary antibodies Alexa Fluor^®^ 488 AffiniPure Goat Anti-Mouse IgG (Jackson ImmunoResearch Laboratories 115-545-146), and Alexa Fluor^®^ 594 AffiniPure Goat Anti-Rabbit IgG (Jackson ImmunoResearch Laboratories 111-585-144) were used in a 1:400 dilution. Samples were incubated with primary antibodies at 4°C for 24 hours. After several PBS washes, secondary antibodies were added and incubated at 4°C for 24 hours. The sections were counterstained with Hoechst 33342 (#14533 Sigma) and imaged with a confocal microscope (ZEISS LSM 880 NLO) or with a fluorescence microscope (Nikon Eclipse E400). A relative quantification of Cx43 staining in Leydig cells was done using ImageJ software. Morphometric evaluations of seminiferous tubule diameter and thickness of the seminiferous epithelium were performed. The diameter of a seminiferous tubule was defined as the shortest distance between two parallel tangent lines of the outer edge of the tubule. Paraffin sections (8 μm) were stained with Periodic acid–Schiff to visualize advanced glycation end products (Periodic acid–Schiff (PAS) kit; 395B-1KT, Sigma).

### Real-Time Reverse-Transcription PCR

RT-qPCR was performed as previously described (29). Briefly, cDNA was prepared using 2 μg of total RNA isolated from the testes. We used RevertAid Reverse Transcriptase (Thermo Fisher Scientific), 1 μL Oligo(dT) and random hexamer primers (Thermo Fisher Scientific) in reverse transcription (RT). Quantitative real-time PCR (qPCR) was performed with a final concentration of cDNA 10 ng/μL using a CFX 384 – qPCR cycler (BioRad). The relative expression of a target gene was calculated, based on the quantification cycle (Cq) difference (Δ) of an experimental sample *versus* control. The control was set at 100% and experimental samples were compared to the control (29). The β-actin (*Actb*) and Peptidylprolyl isomerase A (*Ppia*) genes were used as the reference genes. Primer sequences are listed in **Supplementary Table S1**.

### Statistical Analysis

The comparisons between diabetic and control mice were done using STATISTICA 7.0. (Statsoft, Czech Republic) and GraphPad Prism 7.0 (GraphPad Software, Inc., USA). Differences in organ weights were tested by ANCOVA with body weight as covariate. Differences in sperm parameters, testis morphology, and gene expression between control and T2D groups were assessed by t-test. One-way ANOVA, follow by Tukey’s *post hoc* tests for multiple comparisons was used to assess differences among offspring generations. Repeated measure-based parameters (such as weight gain over time, GTT, or blood glucose levels) were analyzed using two-way ANOVA for repeated measures. *P* value < 0.05 was considered to be statistically significant. Sample sizes and individual statistical results for all analyses are provided in the figure legends and tables.

## Results

### Experimental Paradigm

**Figure 2:**
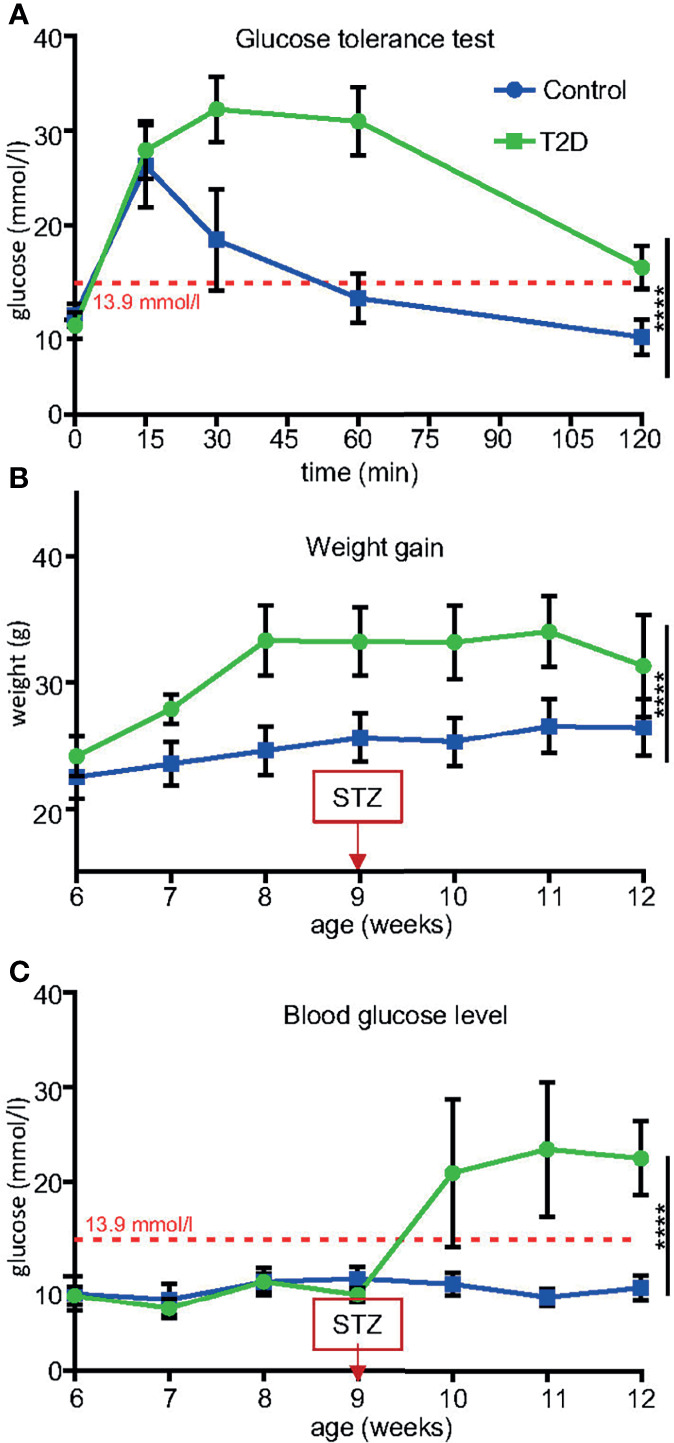
Temporal changes induced in T2D males before mating. **(A)** After 8 weeks of HFD, all males were glucose intolerant compared to control males fed by standard diet. **(B)** Body weight trajectories in T2D males and control males. Note decreased weight gain starting week 11 due to worsening diabetes (hyperglycemia). **(C)** Blood glucose levels significantly increased after a low dose of STZ treatment in combination with HFD, as measured using a glucometer in blood collected from the tail vein after 6-hr fast. Blood glucose levels maintained above 13.9 mmol/l are classified as diabetic (red broken line). Data are presented as the mean ± SD (n = 10 Control and 8 T2D mice). Differences between groups were tested by Two-Way ANOVA for repeated measures, showing significant interactions between time x treatment as repeated measures. ****P < 0.0001.

**Figure 3:**
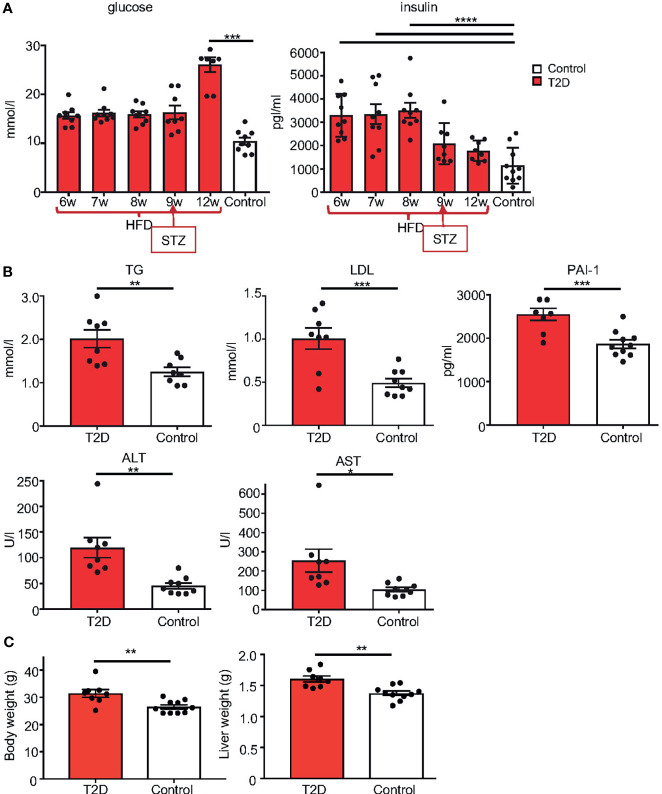
Metabolic phenotype of T2D mouse model. **(A)** Temporal changes in fasting plasma levels of glucose and insulin in T2D males compare to the average level of male controls. Differences were tested by one-way ANOVA with Tukey’s multiple comparisons test. **(B)** Fasting plasma levels of TG, triglycerides; LDL, low-density lipoprotein; marker of insulin resistance, PAI-1, plasminogen activator inhibitor 1; ALT, alanine aminotransferase; and AST, aspartate aminotransferase, in T2D males compared to controls at 12 weeks of age. **(C)** Body and liver weight. Data are presented as the mean ± SEM. Differences between groups were tested by *t*-test (GraphPad Prism 7.0). Differences in liver weight between groups were tested by ANCOVA with body weight as covariate (Ancova – STATISTICA 7.0). *P < 0.05, **P < 0.01, ***P < 0.001, ****P < 0.0001.

We used a non-genetic type 2 diabetes (T2D) mouse model that manifests the metabolic abnormalities associated with human prediabetes and type 2 diabetes (28, 30). This model involves a combination of a high fat diet (HFD) to induce insulin resistance and hyperinsulinemia, and a low dose STZ, which reduces β-cell mass but does not cause diabetes in control-fed mice, to bring about glucose intolerance and hyperglycemia (28, 38) (**Figure 1** Experimental schematics). Together these two stressors have been designed to mimic the pathology and the multi-genetic/environmental background of T2D in humans (39–41). We continuously monitored the metabolic changes in our T2D model. As expected, after 8 weeks of HFD, all male C57BL/6J male mice in the experiment had significantly higher fasting plasma insulin levels and were glucose intolerant, as shown by glucose tolerance tests, indicating compromised insulin response (**Figure 2A**). Body weight of the T2D mice increased progressively over time compared to control mice maintained on a normal standard chow diet (**Figure 2B**). After STZ treatment, mice became hyperglycemic with blood glucose levels maintained above 13.9 mmol/l, classified as diabetic (**Figure 2C**). Fasting plasma levels of glucose and insulin were significantly higher in the T2D group (**Figure 3A**). Correspondingly to the diabetic phenotype, after STZ treatment, plasma glucose levels were increased. Plasma insulin levels, which were initially increased in response to the fat-enriched diet in T2D mice, decreased after STZ-induced β-cell reduction but remained at higher levels than insulin levels in control mice. After 12 weeks, compared to control males, T2D mice displayed significant changes in serum metabolic biomarkers, including increased serum glucose, triglycerides, low-density lipoprotein (LDL), plasminogen activator inhibitor 1 (PAI-1, a marker of insulin resistance), metabolic liver enzymes, alanine aminotransferase (ALT) and aspartate aminotransferase (AST) (**Figure 3B**). Metabolic differences were also manifested by increased body weight, and liver weight of T2D males (**Figure 3C**). These physiological and metabolic changes confirmed decreased insulin sensitivity and impaired glucose tolerance associated with the development of type 2 diabetes mellitus. To analyze the effects of the T2D environment on the reproductive system and reproductive outcomes, we mated these T2D males or control males to female mice maintained on a control chow diet through the course of the experiment (**Figure 1**). After 1 week of mating, males were removed, limiting their influence on their progeny to the mating itself.

### Effects of T2D on the Reproductive Performance, Reproductive Organs, and Sperm Parameters of the Paternal Generation

All diabetic males, with the exception of one mouse, were able to mate in the period of one week. The rate of pregnancy was in the limits of the rate of pregnancy in our C57BL/6J colony (typically 70–80%, unpublished observation), indicating that overall reproductive performance of T2D males was comparable to controls. However, the litter size of T2D males was more variable (5.63 ± 1.35, n = 6 litters) compared to controls (7 ± 0.53, n = 7 litters, **Table 1**). The weights of the reproductive organs, epididymis and seminal vesicles, and the anogenital distance (AGD), as an androgen-responsive outcome, were unaffected in T2D mice, except for the prostate (**Supplementary Table S2**).

**Table 1:** Reproductive effects of T2D in males (parental generation, P).

Group	n	Number of litters	Litter size	Male offspring	Female offspring	Pregnancy rate (%)
**Control**	7	7	7.00 ± 0.53	4.29 ± 0.52	2.71 ± 0.42	100
**T2D**	8	6	5.63 ± 1.35	2.75 ± 0.70	2.88 ± 0.91	75

Data are presented as the mean ± SEM.

**Figure 4:**
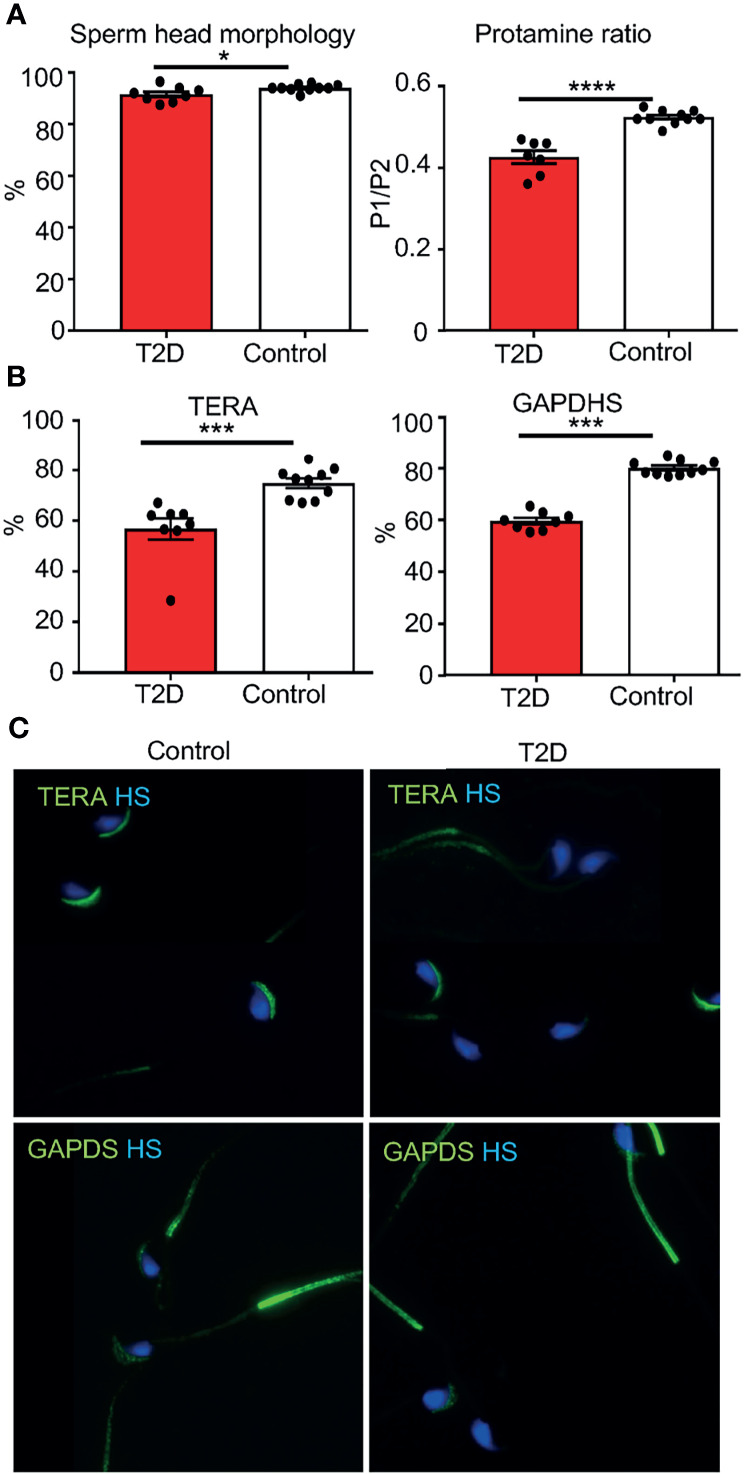
Changes in sperm parameters of the paternal T2D generation. **(A)** Evaluations of caudal epididymal sperm from T2D and control males: abnormalities in the sperm head morphology and protamine 1 and protamine 2 ratios. **(B)** Evaluation of the expression of TERA and GAPDS. **(C)** Representative images of immunolabeling of TERA in the sperm acrosome and GAPDS mainly localized in the principal piece of the flagellum of caudal epidymal sperm from T2D and control males (600x magnification). Data are presented as the mean ± SEM. Differences between groups were tested by *t*-test (GraphPad Prism 7.0). *P < 0.05, ***P < 0.001, ****P < 0.0001. TERA, transitional endoplasmatic reticulum ATPase; GAPDS, glyceraldehyde 3-phosphate dehydrogenase-S.

We performed a sperm quality assessment of collected caudal epididymal sperm. No significant effects were found in sperm concentration, viability, apoptosis, or in the packaging quality of the chromatin (as assessed by chromomycin A_3_ staining) between T2D and control males (**Supplementary Table S3**). The abnormalities in sperm head morphology were increased in T2D males compared to controls (**Figure 4A**). To further assess sperm quality, we analyzed protamine 1 and protamine 2 ratios, which affect DNA stability, and play a role in the establishment of epigenetic marks (42, 43). The protamine 1 and protamine 2 ratios were altered in the paternal T2D generation (**Figure 4A**). Additionally, the expression of biomarkers of sperm dysfunction, transitional endoplasmic reticulum ATPase (TERA) and glyceraldehyde-3-phosphate dehydrogenase-S (GAPDS) was evaluated. TERA is expressed in the acrosomal part of the sperm head (33), whereas GAPDS is located mainly in the principal piece of the sperm flagellum and in lesser amount in the acrosome (34, 44). The expression of both TERA and GAPDS was reduced in sperm from T2D males compared to sperm from control males (**Figures 4B, C** **)**.

### Morphological and Molecular Changes in the Testes of the T2D Paternal Generation

**Figure 5:**
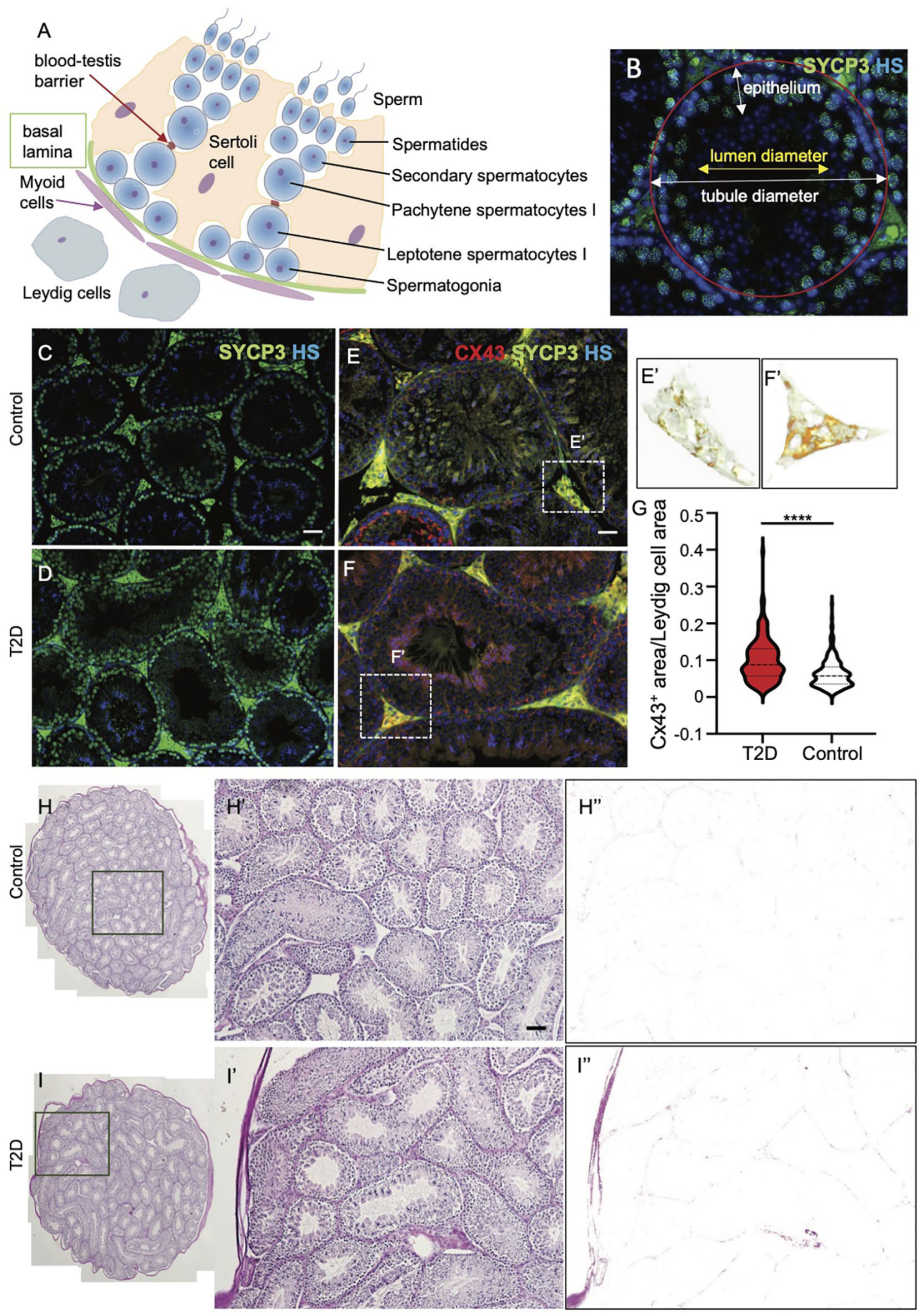
Morphological and expressional changes in the testes of T2D males. **(A)** Schematic cross-section through a testicular tubule, showing germ cells at different stages of spermatogenesis embedded within the somatic Sertoli cells. Leydig cells are present in the interstitium. The blood-testis barrier facilitates the migration of spermatocytes in addition to the maintenance of the microenvironment. **(B)** Morphological evaluation of seminiferous tubules included the measurements of tubule diameter and epithelium thickness for different spermatogenesis stages. **(C, D)** Representative images of cross sections of control and T2D testes after immunolabeling of meiotic marker SYCP3, an axial element of the synaptonemal complex, show the germinal epithelium lining seminiferous tubules at different stages of spermatogenesis. Nuclei are counterstained with Hoechst 33342 (HS, blue). **(E–G)** Immunolabeling of tissue sections shows increased Cx43 expression in the interstitial tissue of the testes of T2D males compared to controls. **(E’, F’)** Delineated Cx43^+^ area in the testis sections by ImageJ program. A relative quantification of staining determined as a ratio of Cx43^+^ areas per the total Leydig cell areas by ImageJ. Stacked violin plot shows the differential expression of Cx43 in control and T2D. Data are presented as the mean ± SEM (n = 6 controls/271 Leydig cell areas, and 6 T2D/198 Leydig cell areas). Differences between groups were tested by *t*-test (GraphPad Prism 7.0). ****P < 0.0001. Nuclei are counterstained with Hoechst 33342 (blue). **(H–I’)** Representative images of PAS staining of the control and T2D testes show increased advanced glycation end-products in the interstitial tissue of the testes from T2D males. **(H”, I”)** Delineated PAS^+^ area in the testes by ImageJ. Scale bars, 50 µm.

In mature testes, sperm are produced in the seminiferous epithelium during the cycle of I to XII stages of spermatogenesis, which are defined by the differentiation steps of germ cells, and occur continuously (45). In order to evaluate the morphology of the seminiferous epithelium at different stages of the differentiation of spermatogenic cells, we focused on four broad categories of the differentiation - spermatogonia, spermatocytes, spermatids, and spermatozoa (Schematic drawing in **Figure 5A**). We did not find any pronounced morphological abnormalities, such as testicular atrophy, deficiency, or loss of the seminiferous epithelium between the control and T2D testes. Immunolabeling of a meiotic marker, synaptonemal complex protein 3 (SYCP3) (46), revealed that T2D testes contained all stages of meiotic cells similar to controls (**Figures 5B–D**). Although all the distinct developmental stages of spermatogenesis were identified in similar percentages in both control and T2D testes, a significantly thinner germinal epithelium was found in all cycle stages in the testes of diabetic mice and the diameter of seminiferous tubules was reduced in diabetic testes compared to controls (**Tables 2**, **3** and **Supplementary Table S4**). To evaluate early markers of diabetes-induced tissue damage, the expression of gap junction protein connexin 43 (Cx43) and production of advanced glycation end products (AGE) in the testes was compared between control and T2D mice. Cx43 levels were increased specifically in the interstitial tissue containing Leydig cells, which are adjacent to the seminiferous tubules in the testes, suggesting altered intercellular communications between neighboring Leydig cells in the T2D testes (**Figures 5E–G**). Leydig cells are the primary source of testosterone or androgens in males, under the regulation of the hypothalamic-pituitary axis, and are vital for spermatogenesis (47). We also found an increased production of AGE in the testicular tissue from T2D males (**Figures 5H–I”**). As AGE are implicated in diabetes related complications, consequently, the formation of AGE may contribute to testicular dysfunction.

**Table 2:** Tubule diameter (μm) for different stages of sperm production in the testes.

Group	I – III	IV – VI	VII – VIII	IX – XII
**Control**	182.781 ± 4.093	186.915 ± 2.415	191.109 ± 2.325	187.220 ± 2.512
**T2D**	164.399 ± 4.852*	176.009 ± 3.039**	174.310 ± 2.609***	167.963 ± 3.437***

Data are presented as the mean ± SEM (n = 10 controls, 206 tubules; n = 8 T2D males, 254 tubules). Differences between groups were tested by t-test (GraphPad Prism 7.0). *P < 0.05, **P < 0.01, ***P < 0.001.

**Table 3:** The seminiferous epithelium thickness (μm) for different stages of sperm production.

Group	I – III	IV – VI	VII – VIII	IX – XII
**Control**	102.465 ± 4.466	105.582 ± 2.859	115.561 ± 2.264	99.791 ± 2.317
**T2D**	73.778 ± 4.230***	90.187 ± 2.885***	108.736 ± 2.438*	84.105 ± 2.555***

Data are presented as the mean ± SEM (n = 10 controls, 206 tubules; 8 T2D males, 254 tubules). Differences between groups were tested by t-test (GraphPad Prism 7.0). *P < 0.05, ***P < 0.001.

**Figure 6:**
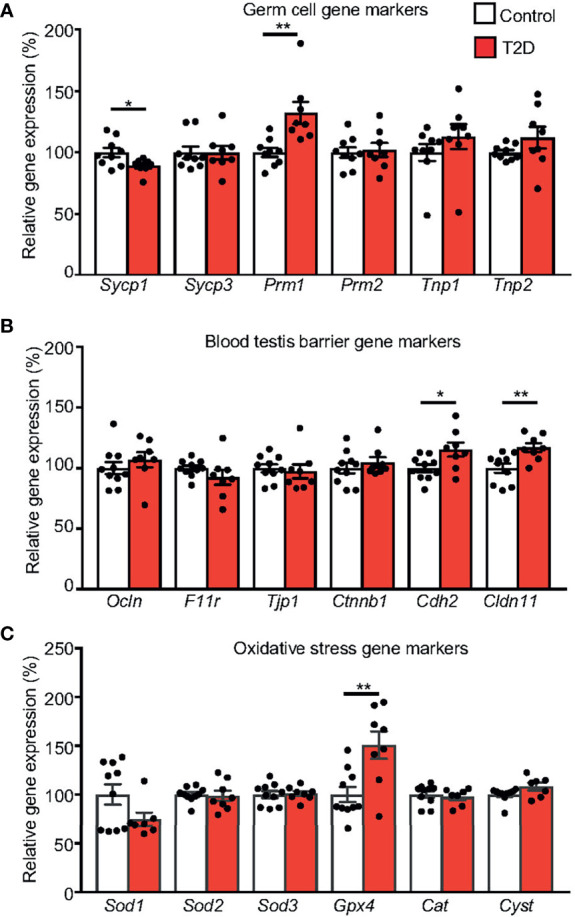
Expression changes in the testes of the parental generation. **(A)** qPCR analysis of markers for spermatogenesis and spermiogenesis: *Sycp1*, synaptonemal complex protein 1; *Sycp3*, synaptonemal complex protein 3; *Prm1*, protamine 1; *Prm1*, protamine 2; *Tnp1*, transition protein 1 and *Tnp2*, transition protein 2; **(B)** Blood testis barrier markers: *Ocln*, occludin; *F11r*, F11 receptor; *Tjp1*, tight junction protein 1; *Ctnnb1*, catenin beta 1; *Cdh2*, cadherin 2; *Cldn11*, claudin 11 and **(C)** oxidative stress markers: *Sod1, Sod2, and Sod3*, superoxide dismutase 1, 2, and 3; *Gpx4*, glutathione peroxidase 4; *Cat*, catalase; and *Cyct*, cytochrome c. The control group represents 100% of relative gene expression. The values are means ± SEM, tested by *t*-test (GraphPad Prism 7.0). *P < 0.05, **P < 0.01.

Having recognized the effects of T2D on testis morphology, we next wanted to assess the effects of the diabetic environment on mRNA expression in testicular tissue. Out of a selection of germ cell marker genes, we found two with altered expression: the expression of *Sycp1* in spermatocytes was reduced and *Prm1* levels were increased correspondingly with changes in the protamine ratio found in sperm from T2D males (**Figure 6A**). We also measured expression of a selection of blood testis barrier markers amongst which we found a significant increase in the expression of *Cldn11*, and *Cdh2* in the testes from T2D males compared to control testes (**Figures 6B, C** **)**.

### Paternal T2D Alters the Metabolic and Reproduction Parameters of Male F_1_ and F_2_ Offspring

To assess whether the paternal response to a diabetic environment affects the reproductive system of male offspring, males on either diet were mated to females. Fathers were removed after mating, limiting influence on their offspring only to the mating itself. The females were housed individually during the gestation period and the litter size was recorded. All females and the offspring generations were fed by the standard, control diet. Only male pups, the F_1_ offspring, were left in the litters to be used for following up transgenerational studies. The F_2_ offspring were produced from the mating of randomly selected F_1_ males with control females (n = 10 males/group). To evaluate paternal effects and minimize any additional environmental influence, all molecular and cellular analyses of both F_1_ and F_2_ offspring were performed at 10 weeks of age, when adult male mice are considered sexually mature (48, 49).

**Figure 7:**
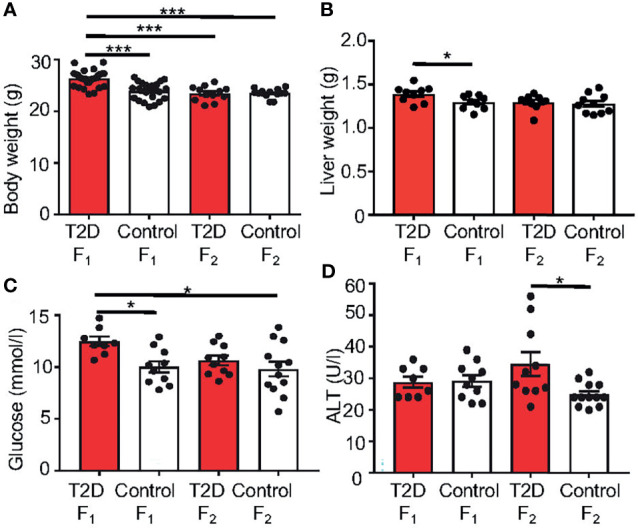
Changes in metabolic characteristics of the offspring generations. **(A)** Body weight and **(B)** liver weight at 10 weeks of age. Differences in liver weight between groups were tested by ANCOVA with body weight as covariate (Ancova – STATISTICA 7.0). **(C)** Fasting plasma glucose and **(D)** alanine aminotransferase (ALT) levels at 10 weeks of age. Data are presented as the mean ± SEM. Differences were tested by one-way ANOVA, follow by Tukey’s *post hoc* tests for multiple comparisons (GraphPad Prism 7.0). *P < 0.05, ***P < 0.001.

First, to determine the potential effect of paternal T2D on the F_1_ and F_2_ generations, we examined physiological and metabolic changes in the offspring. At 10 weeks of age, the body and liver weights were significantly increased in the F_1_ offspring of T2D males (**Figures 7A, B** **)**. Most analyzed serum biomarkers were unaffected in the F_1_ and F_2_ offspring with the exception of glucose and ALT (**Figures 7C, D** **)**. Plasma glucose levels were increased in the F_1_ males at 10 weeks of age, however, these F_1_ males had no impairment of glucose tolerance, as shown by a glucose tolerance test. Interestingly, ALT was increased in F_2_ males, indicating a transgenerational effect of T2D exposure.

Next, we evaluated the effects of paternal T2D on the reproductive system and reproductive parameters of the F_1_ and F_2_ male offspring. The testicular morphology of the F_1_ offspring from control and T2D fathers was comparable, including the thickness of the germinal epithelium and diameter of the seminiferous tubules. Accordingly, the mRNA expression of selected genes in the testes was not different between the F_1_ offspring groups (**Supplementary Figure S1**). However, the weight of testes of the F_1_ offspring from T2D fathers was significantly smaller compared to the control F_1_ offspring (**Supplementary Table S5**). Additionally, significantly reduced fertility was found in the F_1_ males from the T2D parental generation with a pregnancy rate of 63% compared to a 90% pregnancy rate in the F_1_ control generation (**Table 4**). Of eight F_1_ males in the experiment, only five males were able to mate in the period of one week and produce progeny with the average litter size of 4.63 ± 1.41 (n = 5 litters) compared to the F_1_ control cohort with the litter size of 6.44 ± 1.14 (n = 9 litters). The sex ratio of born pups was not significantly different (**Table 4**).

**Table 4:** Reproductive effects of T2D in parental generation on the F_1_ offspring.

Group	n	Number of litters	Litter size	Male offspring	Female offspring	Pregnancy rate (%)
**Control F_1_**	10	9	6.44 ± 1.14	3.56 ± 0.65	2.89 ± 0.59	90
**T2D F_1_**	8	5	4.63 ± 1.41	1.88 ± 0.58	2.75 ± 0.86	63*

Data are presented as the mean ± SEM. Pregnancy rate, Binomial test GraphPad Prism 7. *P <0.05.

### Paternal T2D Alters Sperm Parameters in Two Subsequent Offspring Generations

**Figure 8:**
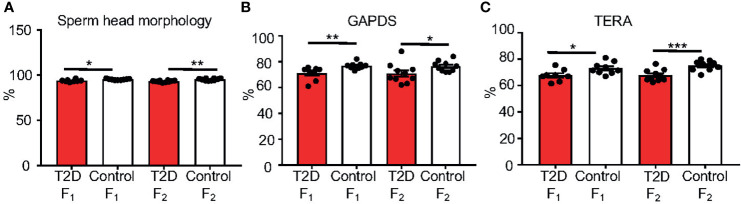
Changes in sperm characteristics of the F_1_ and F_2_ offspring. **(A)** Evaluations of sperm head abnormalities of caudal epididymal sperm from the offspring F_1_ and F_2_ generations from T2D and control fathers. **(B)** The protein levels of glyceraldehyde 3-phosphate dehydrogenase-S (GAPDS) and **(C)** transitional endoplasmatic reticulum ATPase (TERA), as detected by immunolabeling of caudal epidymal sperm from the F_1_ and F_2_ offspring. All analyses were done at 10 weeks of age. Data are presented as the mean ± SEM. (n = 10 for all groups except n = 9 for T2D F_1_ males/200 cells per each animal). Differences between groups were tested by *t*-test (GraphPad Prism 7.0). *P < 0.05, **P < 0.01, ***P < 0.001.

Caudal epididymal sperm were collected from the F_1_ and F_2_ offspring for a sperm quality assessment. Similar to the paternal T2D males, we found no changes in sperm concentration, viability, and cell apoptosis in the F_1_ and F_2_ offspring generations (**Supplementary Table S6**). The packaging quality of the sperm chromatin in the F_1_ and F_2_ offspring was not affected by the T2D parental exposure, as evaluated by chromomycin A_3_ staining of sperm, and by protamine 1 and protamine 2 ratios (**Supplementary Table S6**). However, abnormalities in sperm head morphology were increased in both the F_1_ and F_2_ generations from T2D fathers compared to the control offspring generations (**Figure 8A**). Additionally, the levels of TERA and GAPDS were reduced in the offspring of diabetic males in both F_1_ and F_2_ generations (**Figures 8B, C** **)**, indicating not only intergenerational but also transgenerational transmission of negative effects of the diabetic environment.

## Discussion

In this study, we show that T2D affects not only metabolic health but also the reproductive system through the male lineage to the F_2_ offspring. Paternal T2D exposure causes increased body weight gain and fasting plasma glucose, and significantly impaired reproductive functions of the F_1_ males and sperm abnormalities in the F_1_ male offspring. Furthermore, F_2_ males had altered sperm parameters. Thus, the observed transgenerational non-genetic transmission of sperm damage caused by T2D in fathers indicate programmed sperm perturbations likely in the form of inherited epigenetic marks.

Both fertility and paternally transmitted effects are associated with changes in the sperm epigenome, including non-coding RNAs, DNA methylation, histones, and protamines (28, 50–53). Epigenetic paternal inheritance involving non-coding RNAs has been established in paternal metabolic disorder models. The intergenerational transmission of a paternal HFD-induced metabolic disorder was linked to a subset of sperm transfer RNA-derived small RNAs (tsRNAs) (53). Another study found changes in sperm miRNA profiles and germ cell methylation status in two offspring generations following diet-induced paternal obesity (27). Differential DNA methylation profiles have been identified in multiple regions of the sperm DNA because of environment-induced epigenomic reprogramming (28, 54–56). For example, numerous genes were differently methylated in sperm of prediabetic/T2D fathers, with a significant proportion of differentially methylated genes overlapping with hypermethylated genes in pancreatic islets of the offspring and corresponding to the prediabetes-associated physiological and metabolic phenotype (28). In a model of caloric restriction during *in utero* development, the germline DNA methylation was altered in the adult male offspring (57). Histone variants and histone modifications represent additional modes of paternal transmission of environmental information to the offspring. In contrast to somatic cells, male germ cells have more histone variants and their incorporation into the nucleosome may influence the activation of germline-specific genes and repression of the somatic gene expression program [reviewed in (50, 58)]. Some of the histone variants are exclusively detected on germ cells in testes and seem to also be important in the chromatin condensation process (59). The altered histone modifications may also contribute to transgenerational phenotypes through the male germline, as shown in a transgenic mouse model with histone demethylase KDM1A overexpression, resulting in increased H3K4me3 histone methylation in sperm, a transgenerational phenotype of impaired fertility, and severe birth defects in offspring (51). Similarly, deletion of the H3K4me2 demethylase leads to a transgenerational progressive decline in spermatogenesis (60). Specific histone modifications promote chromatin remodeling and histone-to-protamine replacement, which reshapes the nucleus and compacts chromatin [reviewed in (50, 58)]. The histone-to-protamine transition is a tightly controlled process, as premature expression of protamine 1 or disruption of protamine genes results in male infertility in mice (52, 61). Like histones, protamines have post-translational modifications, which are essential for the histone-to-protamine transitions, and abnormalities in protamine modifications may result in impaired spermiogenesis (62, 63). Epigenetic changes during histone-to-protamine transition may not only lead to reduced fertility but are also transmitted to the offspring (52, 62). Protamine 2-deficient mice demonstrate reduced integrity of sperm DNA, altered compaction of the chromatin, and developmental arrest of embryos (64). Furthermore, alterations in the protamine 1/protamine 2 ratio are related to infertility in humans (43, 65), correlate with worsened assisted reproduction outcomes (66), and aberrant protamine replacement may affect the sperm epigenome [reviewed in (65)]. The role of a ‘protamine code’ in addition to the ‘histone code’ in transgenerational epigenetic inheritance remains to be further investigated. Although adverse metabolic phenotypes correlate with reduced sperm concentration, motility, and increase sperm DNA damage in humans (67–69), a causal relationship by which paternal exposures affect the phenotype of offspring has not been established. Nevertheless, epigenetic changes in sperm are implicated as potential mediators of paternal effects and it is essential to understand how epigenetic patterning is produced and how it is influenced by environmental factors resulting in a greater susceptibility to disease in offspring.

Our study used a well characterized model of type 2 diabetes (28, 30, 70). A combination of high-fat diet and low dose STZ resulted in increased body weight gain, glucose intolerance, and altered metabolic parameters, associated with insulin resistance and hyperglycemia, replicating metabolic changes in T2D patients [reviewed in (71)]. The major advantage of this T2D model is that it provides a more accurate model of the human multi-genetic/environmental T2D phenotype compared to monogenic mouse models, such as the mutant with deficiency in leptin (39, 40) or with deficiency in the leptin receptor (41).

We demonstrated that paternal T2D exposure induces a metabolic shift in glucose and fat metabolism in F_1_ male offspring, as demonstrated by increased plasma glucose together with increased liver weight and body weight. Although the T2D metabolic perturbation phenotype is gradually reversed in the subsequent male generations, increased plasma levels of liver enzyme ALT in the F_2_ males indicate a higher risk for susceptibility to metabolic syndrome and T2D (72, 73).

For the first time, we provide a complex molecular and morphological analysis of the effects of TD2 on the male reproductive system and the effects of TD2 paternal exposure on the reproductive health of two subsequent male offspring generations. Although the testis morphology of T2D mice appeared grossly normal without any atrophy or destruction of the seminiferous epithelium, immunohistological analysis showed a significant reduction of seminiferous tubule diameter and thickness of the seminiferous epithelium for all cycle stages of sperm production in the testes of T2D mice (**Tables 2**, **3**). In line with a lower severity of testicular damage in T2D males, we found only a few expression changes, particularly, altered expression of cell junction proteins, e.g. gap junction protein Cx43 in Leydig cells, and blood-testis-barrier components, tight junction protein, claudin 11 (74), and the adhesion junction component, cadherin 2 (75). Alternations in cell junctions, indicating abnormalities in cell-cell communications, are an early sign of diabetes-induced tissue damage (76). While the F_1_ offspring of T2D males had no detectable molecular and morphological changes in the testes, the weight of testes of the F_1_ offspring of T2D fathers was significantly smaller compared to the control F_1_ males.

A significant finding of this study is the transgenerational transmission of sperm perturbations from T2D fathers through the male lineage to the F_2_ offspring. The direct impact of metabolic diseases, such as obesity and diabetes, on sperm quality in men as well as in animal models is well documented. These negative changes include decreased sperm viability and motility, and increased DNA damage, and sperm morphology abnormalities (67, 77–81). Despite these observations, at present, there is only scant evidence for the impact of paternal exposure on sperm quality and the reproductive viability of subsequent generations. Previously, we showed that type 1 diabetes in male mice induces DNA damage, apoptosis, and alters nuclear protamine ratios in sperm in two subsequent male offspring generations (29). Another study, using a diet-induced obesity mouse model, demonstrated that increased sperm DNA damage and reduced motility was transmitted through the paternal line to the first male offspring generation (82). Similarly, Crisostomo et al. (83) reported irreversible changes in sperm quality in correlation with altered testicular metabolism induced by HFD feeding. Here we focused on the effects of T2D on reproductive health of the young adult offspring. We showed that T2D substantially decreased sperm quality in two subsequent F_1_ and F_2_ offspring male generations. A sperm impairment phenotype consisting of increased sperm head abnormalities and decreased expression of the sperm dysfunctional biomarkers, TERA and GAPDS, resulted in decreased reproductive function of the F_1_ males. Since similar sperm defects were found in the F_2_ offspring, we can speculate that the reproductive function of F_2_ males may also be compromised. TERA is a member of the AAA ATPase family of proteins, and is found in the sperm acrosome (33). Consistent with the role of TERA in capacitation and the acrosome reaction (84), decreased expression of TERA correlates with reduced fertility in humans (33, 35). GAPDS is one of the sperm specific glycolytic enzymes localized primarily in the principal piece of the sperm flagellum and secondary in the acrosomal part of the sperm head (34, 85). It is essential for sperm metabolism, motility and sperm-oocyte binding, and reduced GAPDS is associated with infertility (34, 35, 44, 86).

In conclusion, our data demonstrate that the impaired reproductive system and sperm quality of T2D fathers negatively effects the reproductive health of F_1_ male offspring, which persists into the subsequent F_2_ male generation. Given the transgenerational impairment of reproductive and metabolic parameters through two generations, these changes likely take the form of inherited epigenetic marks through the germline. One possible way could be *via* alterations in protamine 1 and protamine 2 ratios, as we observed changes in this ratio in parental generation. Nevertheless, further investigation of the epigenetic alterations in sperm that result from paternal exposure to T2D are warranted. Consequently, new strategies to improve metabolic health not only in women of reproductive age but also in potential fathers are necessary in order to reduce the negative impacts on subsequent generations.

## Data Availability Statement

The original contributions presented in the study are included in the article/**Supplementary Material**. Further inquiries can be directed to the corresponding author.

## Ethics Statement

The animal study was reviewed and approved by the Animal Care and Use Committee of the Institute of Molecular Genetics, CAS.

## Author Contributions

All authors have read and approved the manuscript. JP and GP conceived the study, designed experiments, and GP wrote the manuscript. FE, HM, and RB conducted animal study, morphological evaluations, and statistical analyses. EZ and EV designed and performed qPCR. SH and AK performed sperm analyses and Western blotting. HM, EZ, and RB performed immunohistological evaluations. All authors contributed to the article and approved the submitted version.

## Funding

This work was supported by the institutional support of the Czech Academy of Sciences RVO: 86652036 and the Czech Health Research Council #NU20J-02-00035.

## Conflict of Interest

The authors declare that the research was conducted in the absence of any commercial or financial relationships that could be construed as a potential conflict of interest.

## Publisher’s Note

All claims expressed in this article are solely those of the authors and do not necessarily represent those of their affiliated organizations, or those of the publisher, the editors and the reviewers. Any product that may be evaluated in this article, or claim that may be made by its manufacturer, is not guaranteed or endorsed by the publisher.
