# Supplementary material for: The Transgenerational Transmission of the Paternal Type 2 Diabetes-Induced Subfertility Phenotype
Source: Front Endocrinol (Lausanne). 2021 Nov 5;12:763863. doi: 10.3389/fendo.2021.763863 (PMC8602877; doi:10.3389/fendo.2021.763863)
Supplement: Supplementary file 1 [file DataSheet_1.pdf]

# Supplementary Material

## 1 Supplementary Figures and Tables

### Supplementary Table S1. List of primer sequences

| Gene symbol | Gene name | RefSeq ID | Primer sequence | |
|---|---|---|---|---|
| *Cat* | catalase | NM_009804.2 | F | 5' -TCACTCAGGTGCGGACATTC - 3' |
| | | | R | 5' -TAGTCAGGGTGGACGTCAGT - 3' |
| *Cdh2* | cadherin 2 | NM_007664.5 | F | 5' -CTTGCTTCAGGCGTCTGTGGA - 3' |
| | | | R | 5' - TTCGTGCACATCCTTCGGTAA- 3' |
| *Cldn11* | Claudin 11 | NM_016674.4 | F | 5' -GGCGACATTAGTGGCCACAGCA- 3' |
| | | | R | 5' -GCAGCGGCCCAGCCAGTAAA- 3' |
| *Ctnnb1* | catenin beta 1 | NM_007614.3 | F | 5' - CGCCGCTTATAAATCGCTCC- 3' |
| | | | R | 5' - TTCACAGGACACGAGCTGAC- 3' |
| *Cyct* | cytochrome c, testis | NM_009989.3 | F | 5' -CGGCTGCTGTGATTGTGAATAC - 3' |
| | | | R | 5' -TGTCTTGTGTTTCCCGCCTT - 3' |
| *F11r* | F11 receptor | NM_172647.2 | F | 5' - AGGTCATTTACAGCCAGCCC- 3' |
| | | | R | 5' -GAAGGCATCCTGTGCAGCTA - 3' |
| *Gpx4* | glutathione peroxidase 4 | NM_001037741.3 | F | 5' - TGTGTAAATGGGGACGATGCC- 3' |
| | | | R | 5' - TCTCTATCACCTGGGGCTCCT- 3' |
| *Ocln* | occludin | NM_008756.2 | F | 5' - CTTATCTTGGGAGCCTGGACAT- 3' |
| | | | R | 5' - ATGCATCTCTCCGCCATACA- 3' |
| *Prm1* | protamine 1 | NM_013637.4 | F | 5' -ACAGGTTGGCTGGCTCGACC- 3' |
| | | | R | 5' -CGGCAGCATCGGTATCTGGCC- 3' |
| *Prm2* | protamine 2 | NM_008933.1 | F | 5' -CCAGGGGCCTGGACAAGACC- 3' |
| | | | R | 5' -TCTGTGGTGGTGGTGGCCCC- 3' |
| *Sod1* | superoxide dismutase 1 | NM_011434.1 | F | 5' - GGAACCATCCACTTCGAGCA- 3' |
| | | | R | 5' -CTGCACTGGTACAGCCTTGT - 3' |
| *Sod2* | superoxide dismutase 2 | NM_013671.3 | F | 5' - GAACAATCTCAACGCCACCG- 3' |
| | | | R | 5' - CCAGCAACTCTCCTTTGGGTT- 3' |
| *Sod3* | superoxide dismutase 3 | NM_011435.3 | F | 5' -CTGACAGGTGCAGAGAACCTC - 3' |
| | | | R | 5' - GCGTGTCGCCTATCTTCTCA- 3' |
| *Sycp1* | synaptonemal complex protein 1 | NM_011516.2 | F | 5' -GCCCATGCTCGAACAG GTTGC- 3' |
| | | | R | 5' -ACAGTCTGCTCATTGGCTCTGAA- 3' |
| *Sycp3* | synaptonemal complex protein 3 | NM_011517.2 | F | 5' -GGACAGCGACAGCTCACCGG- 3' |
| | | | R | 5' -GGTGGCTTCCCAGATTTCCCAGA- 3' |
| *Tjp1* | tight junction protein 1 | NM_009386.2 | F | 5' -TCTTGCAAAGTATCCCTTCTGT - 3' |
| | | | R | 5' -CAGAAATCGTGCTGATGTGCC - 3' |
| *Tnp1* | transition protein 1 | NM_009407.2 | F | 5' -CCGAGCTCCTCACAAG GGCGT- 3' |
| | | | R | 5' -CAGGGCAGAGCTCATTGCCGC- 3' |
| *Tnp2* | transition protein 2 | NM_013694.4 | F | 5' -CCTGCAAGACCCAGCCACCG- 3' |
| | | | R | 5' -GTTTCCGCCTCCTGACGGCC- 3' |

**Table S2.** Anogenital distance and organ weights (parental generation, P)

| Group | n | AGD (cm) | Testes (g) | Epididymides (g) | Prostate (g) | Seminal vesicles (g) | Spleen (g) |
|---|---|---|---|---|---|---|---|
| **Control** | 10 | 1.29±0.01 | 0.209±0.004 | 0.066±0.001 | 0.096±0.003 | 0.273±0.011 | 0.084±0.003 |
| **T2D** | 8 | 1.25±0.01 | 0.207±0.004 | 0.063±0.001 | 0.081±0.004** | 0.244±0.012 | 0.076±0.003 |

Data are presented as the mean ± SEM. Differences between groups were tested by ANCOVA with body weight as covariate (Ancova – STATISTICA 7.0). **P<0.01. AGD, anogenital distance.

**Table S3.** Sperm parameters (parental generation, P)

| Group | n | Concentration (1 * $10^6$/ml) | Viability (%) | CMA3 (%) | Annexin V (%) |
|---|---|---|---|---|---|
| **Control** | 10 | 22.20±2.31 | 80.94±1.23 | 97.50±0.34 | 53.15±1.93 |
| **T2D** | 8 | 16.16±1.90 | 83.58±1.77 | 98.19±0.37 | 57.06±3.42 |

Data are presented as the mean ± SEM. Differences between groups were tested by *t*-test (GraphPad Prism 7.0). CMA3, chromomycin A3

**Table S4.** Representation of the stages of the sperm production in the seminiferous epithelium

| Group | **I – III** (%) | **IV – VI** (%) | **VII – VIII** (%) | **IX – XII** (%) |
|---|---|---|---|---|
| **Control** | 10.41±2.27 | 23.50±1.95 | 30.23±2.16 | 35.86±5.35 |
| **T2D** | 15.73±4.45 | 25.79±3.06 | 27.93±5.33 | 30.54±2.73 |

Data are presented as the mean ± SEM (n = 10 controls, 206 tubules; 8 T2D males, 254 tubules).

**Table S5.** Anogenital distance and organ weights (the offspring generations, $F_1$ and $F_2$)

| Group | n | AGD (cm) | Testes (g) | Epididymides (g) | Prostate (g) | Seminal vesicles (g) | Spleen (g) |
|---|---|---|---|---|---|---|---|
| **Control $F_1$** | 10 | 1.21±0.03 | 0.186±0.003 | 0.057±0.002 | 0.075±0.005 | 0.167±0.008 | 0.101±0.004 |
| **T2D $F_1$** | 9 | 1.22±0.03 | 0.176±0.002** | 0.057±0.002 | 0.087±0.005 | 0.160±0.007 | 0.091±0.004 |
| **Control $F_2$** | 10 | 1.24±0.02 | 0.171±0.009 | 0.054±0.002 | 0.080±0.005 | 0.162±0.010 | 0.093±0.005 |
| **T2D $F_2$** | 10 | 1.22±0.02 | 0.164±0.008 | 0.055±0.002 | 0.083±0.004 | 0.170±0.010 | 0.081±0.005 |

Data are presented as the mean ± SEM. Differences between groups were tested by ANCOVA with body weight as covariate (Ancova – STATISTICA 7.0). **P<0.01. AGD, anogenital distance.

**Table S6.** Sperm parameters (the offspring generations, $F_1$ and $F_2$)

| Group | n | Concetration (1 * $10^6$/ml) | Viability (%) | CMA 3 (%) | Protamine ratio (P1/P2) | Annexin V (%) |
|---|---|---|---|---|---|---|
| **Control $F_1$** | 10 | 14.53±1.90 | 80.98±0.81 | 97.63±0.53 | 0.50±0.01 | 54.69±2.06 |
| **T2D $F_1$** | 9 | 12.94±1.15 | 83.43±1.09 | 98.31±0.35 | 0.50±0.02 | 44.38±1.84 |
| **Control $F_2$** | 10 | 15.45±1.74 | 82.43±1.82 | 98.53±0.27 | 0.53±0.02 | 54.50±2.31 |
| **T2D $F_2$** | 10 | 16.29±1.63 | 83.32±1.45 | 98.05±0.40 | 0.51±0.02 | 48.30±1.58 |

Data are presented as the mean ± SEM. Differences between groups were tested by *t*-test (GraphPad Prism 7.0). CMA3, chromomycin A3

**Supplementary Figures**

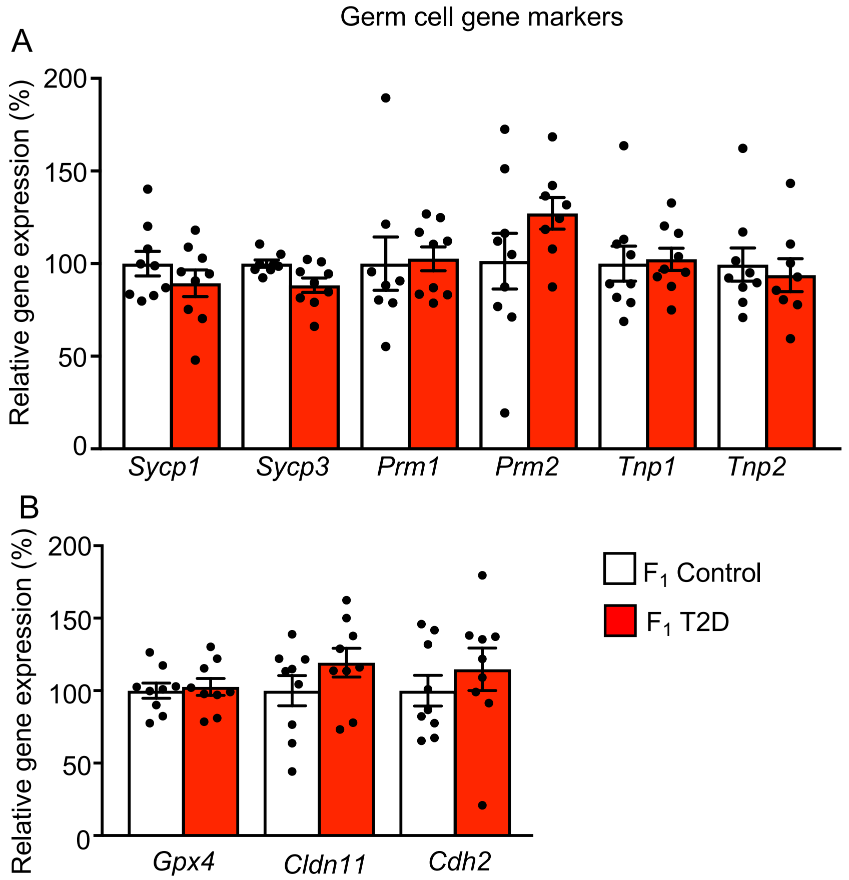


**Supplementary Figure S1. Relative expression of selected genes in the testes of the $F_1$ generation.** qPCR analysis of markers for **(A)** spermatogenesis and spermiogenesis: synaptonemal complex protein 1 (*Sycp1*), synaptonemal complex protein 3 (*Sycp3*), protamine 1 (*Prm1*), protamine 2 (*Prm1*), transition protein 1 (*Tnp1*) and transition protein 2 (*Tnp2*); **(B)** blood testis barrier: cadherin 2 (*Cdh2*), claudin 11 (*Cldn11*); and oxidative stress: glutathione peroxidase 4 (*Gpx4*). The control group represents 100% of relative gene expression. The values are means ± SEM, tested by *t*-test.
